# 2220: Pharmacogenomic determinants in Caribbean Hispanics of clopidogrel failure in acute coronary syndrome

**DOI:** 10.1017/cts.2017.99

**Published:** 2018-05-10

**Authors:** Kyle Melin, Jorge Duconge, Dagmar F. Hernandez Suarez

**Affiliations:** University of Puerto Rico-Medical Sciences Campus, San Juan, Puerto Rico

## Abstract

OBJECTIVES/SPECIFIC AIMS: The objective of this study is to measure the association of CYP2C19 (*1-*8,*17), ABCB1(C3435T; rs1045642), PON1 (p.Q192R; rs662), and B4GALT2 (c.909 C>T and c.366 G>C) gene polymorphisms in the Caribbean Hispanic population with major adverse cardiovascular events (MACE). METHODS/STUDY POPULATION: Patients of Caribbean Hispanic ethnicity from all geographic regions of the Island of Puerto Rico, male and female, aged >21 will be recruited. Cases will consist of patients receiving a daily clopidogrel dose of 75 mg following acute coronary syndrome (ACS) who experience a MACE within the first year of treatment. Control study patients must have received clopidogrel 75 mg daily for a minimum of 1 year without experiencing MACE. Genomic DNA samples will be genotyped to determine the frequency distribution of major CYP2C19, ABCB1, PON1, and B4GALT2 gene polymorphisms. Observed frequencies will be compared with other reported populations. An association study will be performed between genetic variables and MACE and a multivariable logistic regression model (additive) will be constructed. RESULTS/ANTICIPATED RESULTS: We anticipate finding a significant association between major genetic determinants of clopidogrel response and MACE where cases with MACE will carry higher frequency of CYP2C19, ABCB1, PON1, and B4GALT2. DISCUSSION/SIGNIFICANCE OF IMPACT: As the range of multiloci allelic combinations in admixed Caribbean Hispanics is higher than in other populations due to its unique 500-year history of genomic admixture, a wide spectrum of genetic variances is expected to be present in the study population. Determining the prevalence and effect of CYP2C19, ABCB1, PON1, and B4GALT2 polymorphisms holds the potential to personalize anti-platelet treatment for Caribbean Hispanic patients requiring treatment after ACS.

